# Region-wise analysis of dairy cow movements in Japan

**DOI:** 10.1186/s12917-021-03008-3

**Published:** 2021-09-09

**Authors:** Yoshinori Murato, Yoko Hayama, Yumiko Shimizu, Kotaro Sawai, Emi Yamaguchi, Takehisa Yamamoto

**Affiliations:** Epidemiology Unit, National Institute of Animal Health, National Agriculture and Food Research Organization, Tsukuba, Ibaraki Japan

**Keywords:** Animal infectious diseases, Cattle movement, Japan, Traceability system

## Abstract

**Background:**

Animal movement is considered the most significant factor in the transmission of infectious diseases in livestock. A better understanding of its effects would help provide a more reliable estimation of the disease spread and help develop effective control measures. If the movement pattern is heterogeneous, its characteristics should be considered in epidemiological analyses, such as when using simulation models to obtain reliable outputs. In Japan, following the bovine spongiform encephalopathy epidemic, a traceability system for cattle was established in 2003, and the registration of all cattle movements in the national database began. This study is the first to analyze cattle movements in Japan. We examined regional and seasonal heterogeneity in dairy cow movements, which accounted for most Japanese breeding cattle.

**Results:**

In the 14 years from April 2005 to March 2018, 4,577,709 between-farm movements of dairy cows were recorded, and the number of movements was counted by month and age for both inter- and intra-regional movements. As a result, two characteristic round-trip movements were observed: one was non-seasonal and inter-regional movements related to cattle-breeding ranches in Hokkaido (the northern region of Japan), which consists of the movement of cows around ages 6 to 8 and 21 to 23 months old. In addition, the seasonal movement of heifers for summer grazing within Hokkaido occurred in May and October at the peak ages of 13 to 14 and 19 to 20 months old, respectively. The observed heterogeneity seemed to reflect the suitability of raising the Holstein breed in Hokkaido and the shortage of supply of replacement heifers and available farming areas outside Hokkaido.

**Conclusions:**

Understanding the patterns of dairy cow movements will help develop reliable infectious disease models and be beneficial for developing effective control measures against these diseases.

**Supplementary Information:**

The online version contains supplementary material available at 10.1186/s12917-021-03008-3.

## Background

The movement of animals is one of the most important routes of infectious disease transmission in livestock. In the case of the foot and mouth disease outbreak in the United Kingdom in 2001, the movement of infected animals, before the movement ban, was recognized as a major contributor to the nationwide spread of the disease [1]. Meanwhile, in the case of infectious diseases with a long incubation period, such as bovine tuberculosis, the movement of infected cattle is also known to contribute to the disease transmission between farms without alerting farmers of the current situation [2, 3]. Thus, to develop efficient control measures against diseases, sufficient knowledge of animal movement patterns is a prerequisite.

In addition, as infectious disease modeling becomes a more popular technique for predicting the spread of diseases and evaluating the effectiveness of control measures, the use of a more realistic animal movement pattern is important. If the movement of animals is considered heterogeneous, the movement characteristics should be properly considered in the models to obtain reliable outputs. Therefore, animal movement characteristics have been analyzed and reported in some countries such as the UK and Australia [4–7]. However, animal movement patterns will be subject to various influences, such as geographical, environmental, economic, and biological factors.

In Japan, following the outbreak of bovine spongiform encephalopathy (BSE) in 2001, a traceability system for cattle was established; registration of the individual identification number and reporting all cattle movements to the national database became a legal requirement [8]. Since this traceability system has been in place for more than a decade and an adequate amount of data has been collected, the analysis of cattle movement has become possible.

Japan is an island country approximately 3,000 km in length, straddling subarctic and subtropical zones. As of February 2020, 3.91 million cattle have been raised in Japan, of which 1.35 million are dairy cows (including heifers and calves), accounting for 1/3 of all cattle. Of these, 622,000 are beef cattle for breeding (mostly Wagyu cattle), and the remaining are beef feedlot cattle, including male dairy cattle. Holstein is the primary dairy breed in Japan, and the major beef breed is Japanese Black, and each breed has its optimal climate. The upper critical temperature for the Holsteins is lower than that of the Japanese Black [9, 10]. High temperatures are unsuitable for Holsteins; therefore, 60 % of dairy cows are raised in Hokkaido, the northern island of Japan, and 40 % of the beef cattle breed, which are relatively heat-tolerant, are raised in Kyushu, the southern island of Japan. This climatic and geographic diversity, as well as breed characteristics, may cause regional heterogeneity in cattle farming. Thus, cattle movements are expected to be regionally heterogeneous.

**Fig. 1:**
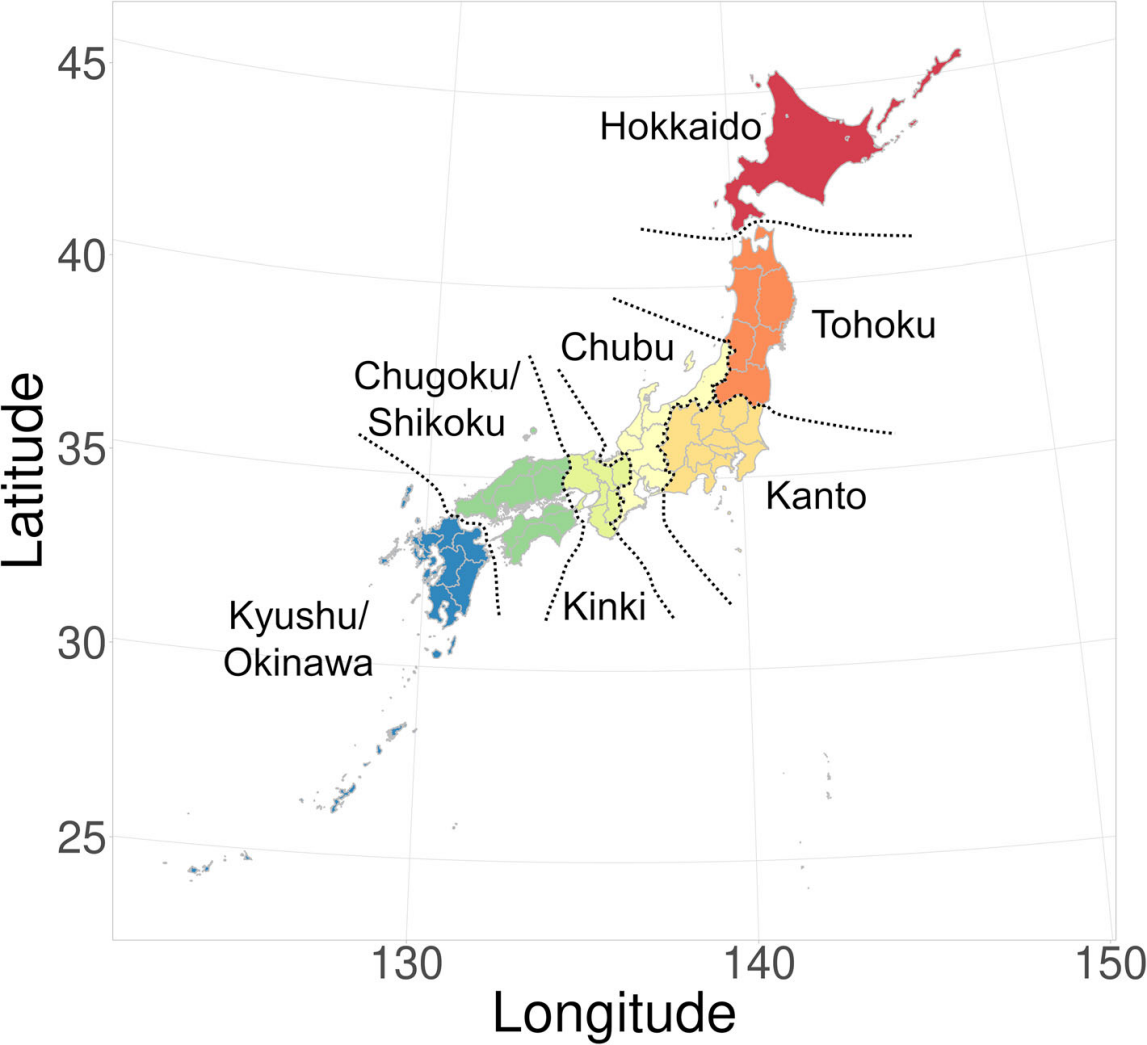
Classification of Japanese regions in this study

This study examined the geographical and seasonal heterogeneity of cattle movement in Japan to assist with the development of control measures or modeling studies. In this work, we focused on dairy cows, which form the majority (68 %) of breeding cattle and are expected to reflect the complexity of cattle movement in Japan. We have descriptively analyzed the characteristics of movement patterns at the regional level (the classification of regions is shown in Table 1; Fig. 1).

**Table 1:** Classification of Japanese regions in this study

No.	Name of Region	Abbreviation of Region
1	Hokkaido	HKD
2	Tohoku	THK
3	Kanto	KTO
4	Chubu	CHU
5	Kinki	KNK
6	Chugoku/Shikoku	C_S
7	Kyushu/Okinawa	K_O

## Results

### Overview of the dairy cow population in Japan

The number of dairy cows by region as of April 1, 2018, is shown in Figure S1 (Additional file 1); 1.37 million dairy cows of all ages were raised in Japan, of which 819,000 cows (60 %) were kept in Hokkaido. The number of dairy cows born during FY2018 (FY means the Japanese fiscal year, which starts April 1 and ends March 31 of the following year) by region is shown in Figure S2 (Additional file 1); the monthly births are shown in Figure S3 (Additional file 1). A total of 271,000 dairy cows were born in Japan in FY2018, of which 187,000 cows (68 %) were bred in Hokkaido.

### Overview of between-farm movement

**Fig. 2:**
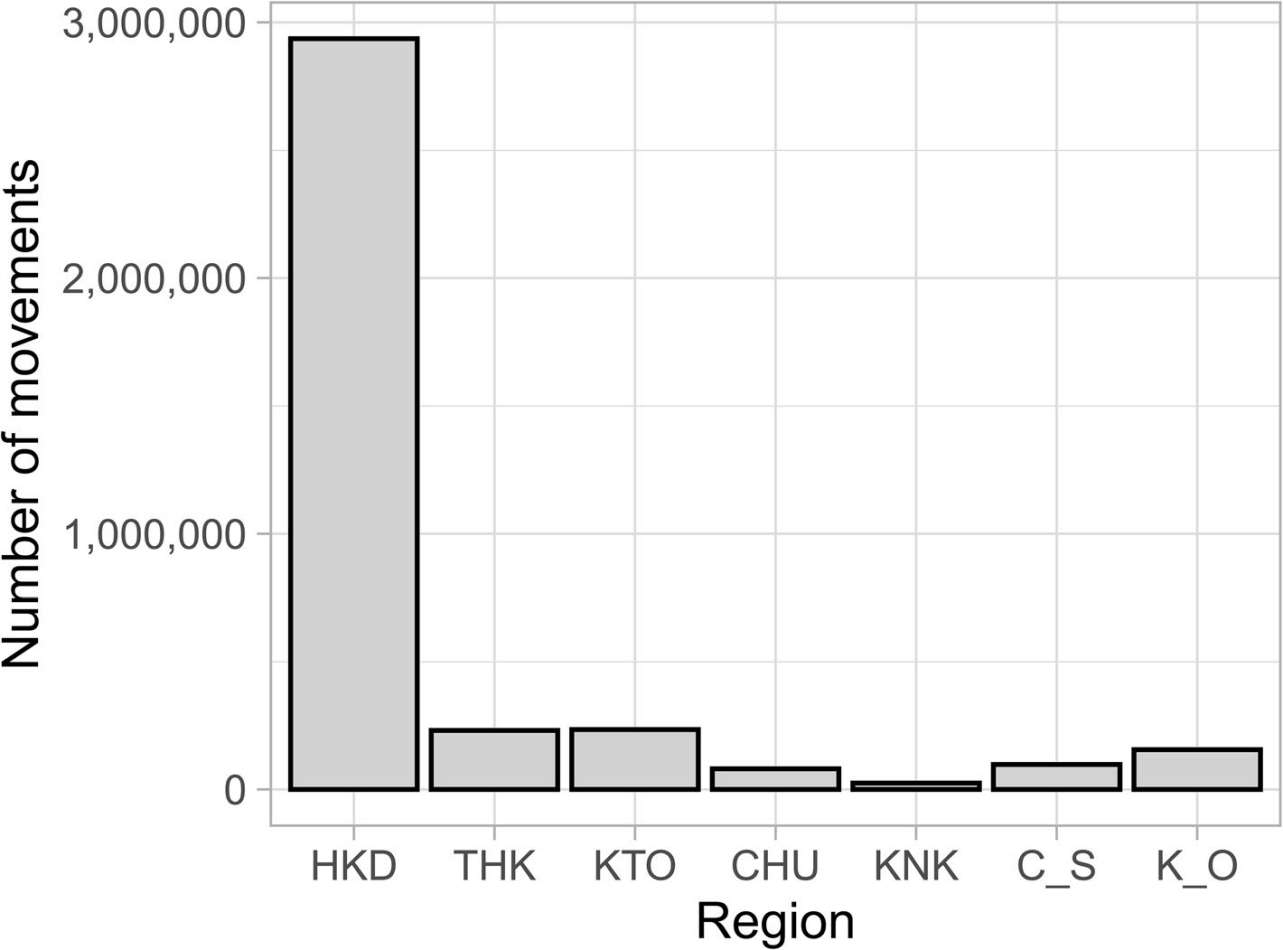
Number of intra-regional movements of dairy cows by region in Japan from FY2005 to FY2018

**Fig. 3:**
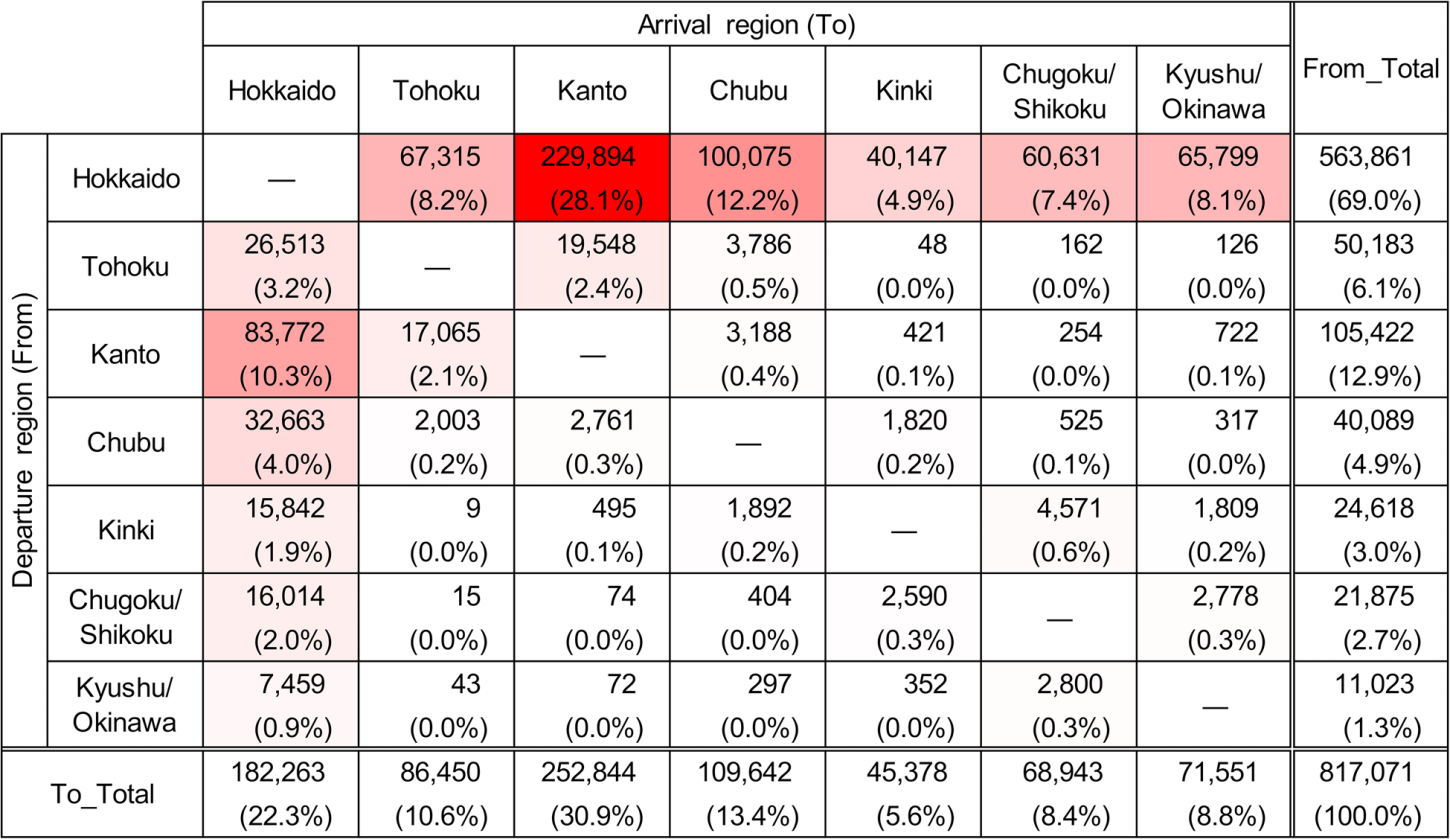
Number of inter-regional movements of dairy cows. The number of movements by dairy cows, between each departure and arrival region in Japan from FY2005 to FY2018. Gradation of red shades in the background to the cells indicates higher numbers

In the 14 years from FY2005 to FY2018, 4,577,709 between-farm movements of dairy cows were recorded. The average number of movements per year was 327,000 (300,414 to 366,541) and was generally similar across the 14 years (Additional file 1: Figure S4). On the other hand, 3,760,638 movements, accounting for 82 % of all between-farm movements, were intra-regional movements, and the remaining 18 % (817,071 movements) were inter-regional movements. Of the former, 78 % were within Hokkaido (Fig. 2), and 91 % of the latter departed from or arrived in Hokkaido (Fig. 3).

### Inter-regional movement

**Fig. 4:**
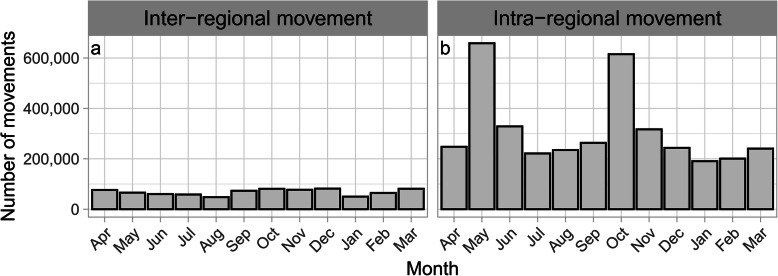
Number of monthly movements of dairy cows. Inter-regional (**a**) and intra-regional movements (**b**) of dairy cows in Japan from FY2005 to FY2018

**Fig. 5:**
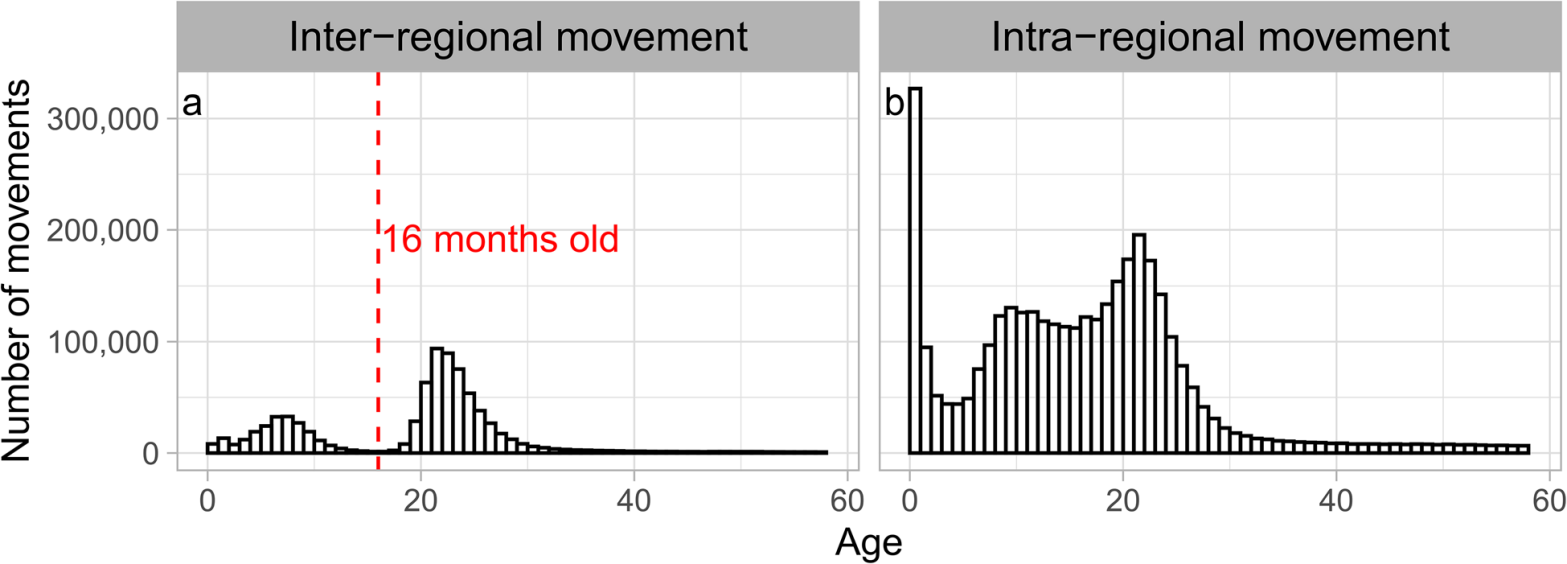
Age distribution at the movement of dairy cows. In inter-regional (**a**) and intra-regional movements (**b**) of dairy cows in Japan from FY2005 to FY2018

**Fig. 6:**
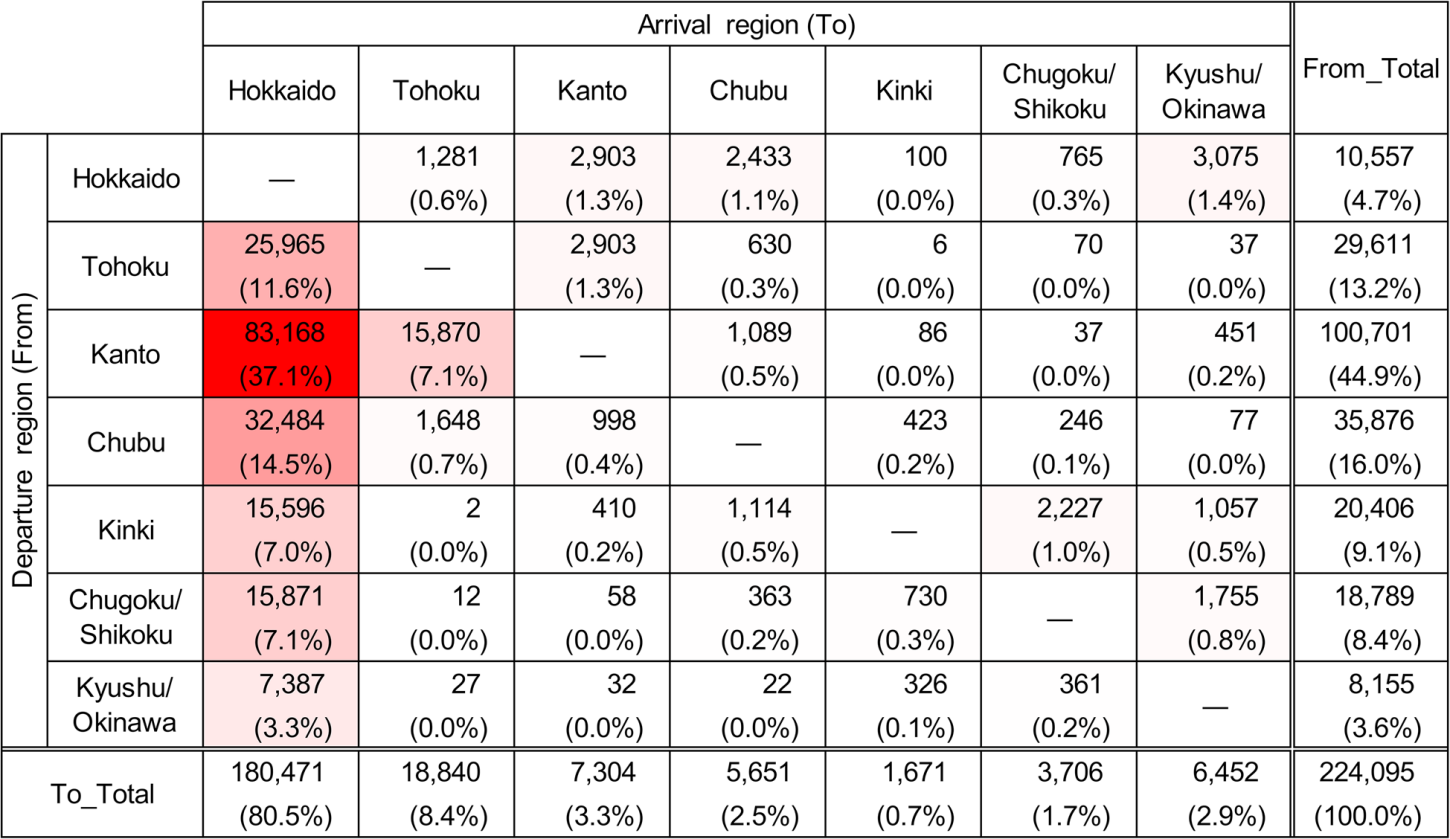
Number of inter-regional movements of dairy cows under 16 months old. Movements between each departure and arrival region in Japan from FY2005 to FY2018 are shown. Gradation of red shades in the background to the cells indicates higher numbers

**Fig. 7:**
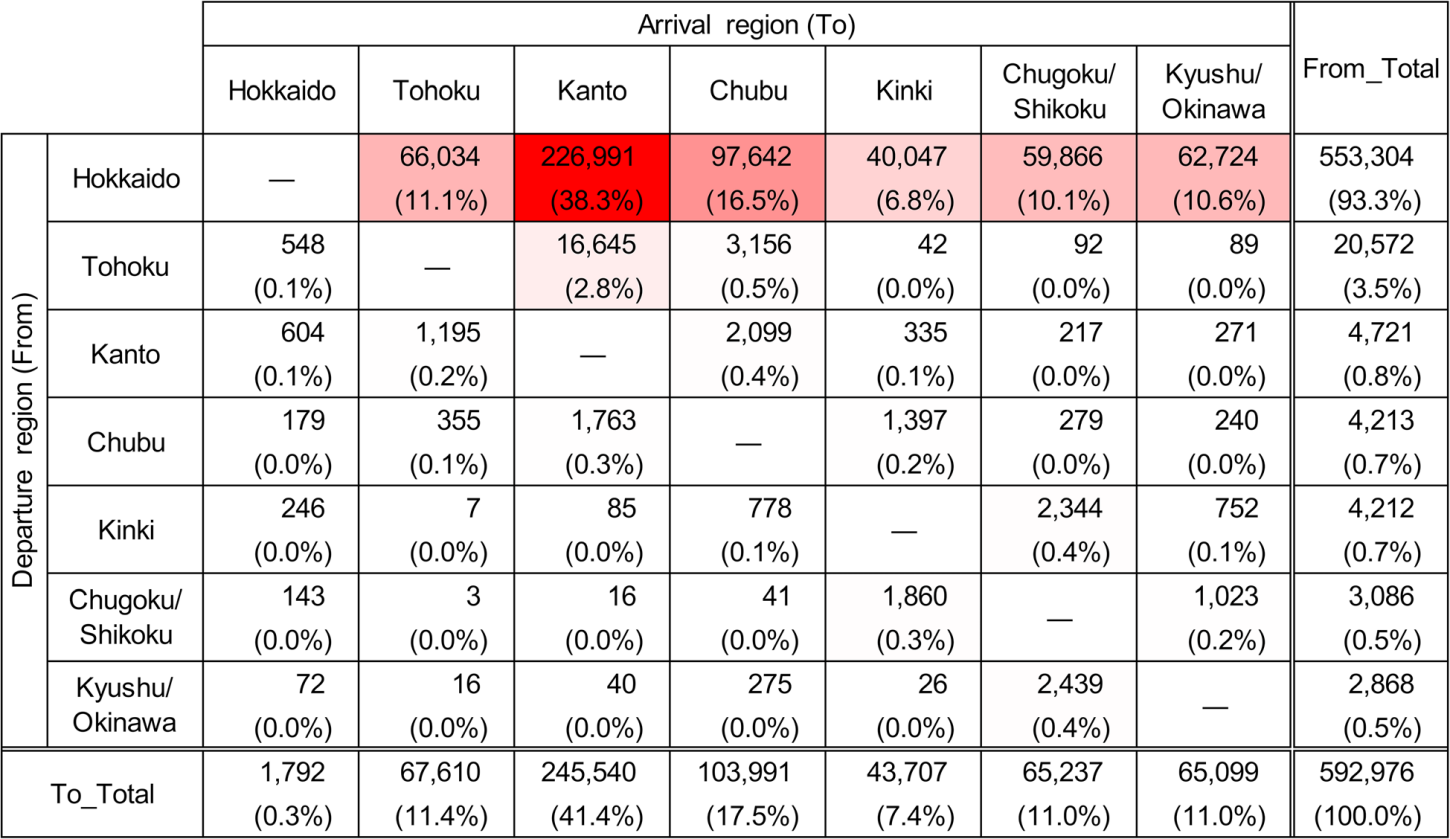
Number of inter-regional movements of dairy cows over 16 months old. Movements between each departure and arrival region in Japan from FY2005 to FY2018 are shown. Gradation of red shades in the background to the cells indicates higher numbers

The number of monthly inter-regional movements was almost constant (Fig. 4a). The age distribution at movement was bimodal, with peaks at 6 to 8 and 21 to 23 months and a trough at 15 to 17 months of age (Fig. 5a). Since the age distribution at movement formed two groups that were centered at each peak of the bimodal distribution, the data were divided into 2 groups at 16 months for further analysis. The number of inter-regional movements of cows under 16 months old was 224,095, and out of the total, 180,471 movements (81 %) arrived in Hokkaido (Fig. 6). Cows over 16 months old moved 592,976 times, out of which, 553,304 movements (93 %) departed from Hokkaido (Fig. 7), and 89 % of them were less than 30 months old. Of the post-16-month-old cow movements departing from Hokkaido, 136,916 (25 %) were return movements of dairy cows shipped from other regions when under 16 months of age.

### Intra-regional movement

**Fig. 8:**
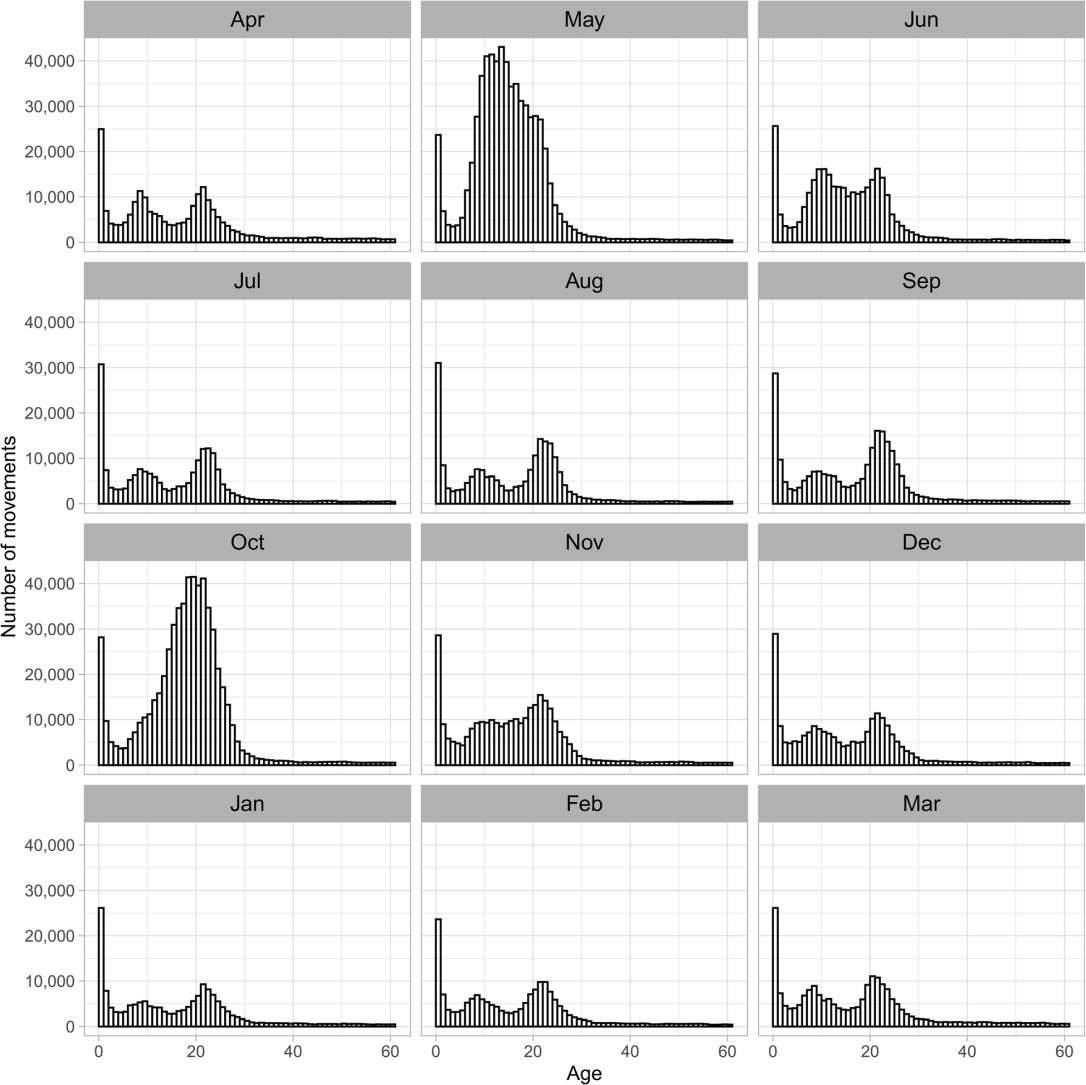
Age distribution of dairy cows at intra-regional movements in Japan from FY2005 to FY2018. Monthly data are shown

**Fig. 9:**
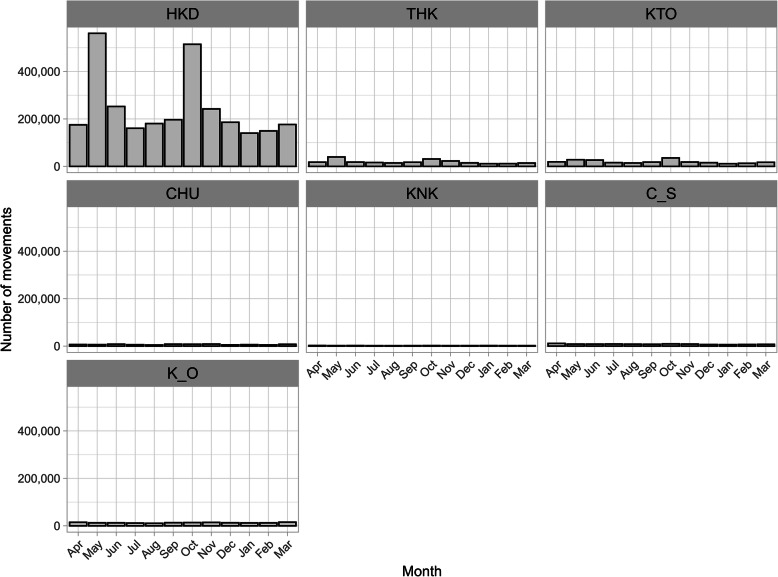
Number of intra-regional movements of dairy cows in Japan from FY2005 to FY2018. Monthly data are shown

The number of monthly Intra-regional movements was higher in May (18 %) and October (16 %) than in other months (5 to 9 %) (Fig. 4b). This seasonality was observed throughout the study period (Additional file 1: Figure S5). The age distribution at movement was trimodal, with peaks at less than 1 month, 9 to 10 months, and 21 to 22 months (Fig. 5b). The number of movements varied monthly; therefore, the age distribution at movement was compared by month. In May and October, the age distribution at movement differed from that in other months (Fig. 8). The age distribution in months at movement was trimodal, except for May and October, with peaks at less than 1 month old, around 9 months old, and around 22 months old (Fig. 8). As observed in other months, both the age distributions in May and October peaked at less than 1 month of age. In addition, a peak at 13 to 14 months of age was observed in May, and a peak at 19 to 20 months of age was observed in October (Fig. 8). Compared with the number of monthly movements by region, Hokkaido showed peaks in May and October, and Tohoku and Kanto showed a slightly similar trend (Fig. 9).

Since the peaks of age at movement, exceptionally observed in May and October, showed an almost 6-month gap, equivalent to the time difference between May and October, it was suggested that the data reflected the round trips of the same cows. Hence, to examine this hypothesis, we analyzed movement at the individual level using FY2018 data. Consequently, 37 % of the cows that moved in May also moved in October, accounting for 40 % of the cows that moved in October. As for these round-trip cows, the age at movement peaked at 13 to 14 months in May and 19 to 20 months in October.

## Discussion

This study is the first to analyze the movement of cattle in Japan. Dairy cow movements were analyzed descriptively to reveal their characteristics at the regional level. As a result, movement heterogeneity was observed, and the movement pattern varied depending on age, region, and season.

Regarding inter-regional movements, Hokkaido was the major importer, as well as the major exporter. 91 % of the inter-regional movement of cattle departed from or arrived in Hokkaido (Fig. 3). 81 % of the pre-16-months-old movements arrived in Hokkaido (Fig. 6), and 93 % of the post-16-months-old movements departed from Hokkaido (Fig. 7). Thus, these movements can be interpreted as round trips centered on Hokkaido, importing cows around 6 to 8 months old, and exporting cows around 21 to 23 months old. Considering the suitable breeding age (around 14 months old) of the Holstein breed, most dairy cows shipped to Hokkaido could be pre-breeding calves. Then, the dairy cows shipped from Hokkaido aged 21 to 23 months could represent the export of pregnant heifers after breeding operations conducted at breeding ranches in Hokkaido. These heifers’ returning age agrees with the average age of the first calving in dairy cows (24.7 months old) in Japan [11], as they will give birth at farms outside Hokkaido, then followed by milking. The highest number of movements into and from Hokkaido were linked to Kanto (forward: 37.1 %, backward: 38.3 %) and this might be explained by the highest number of dairy cattle raised in Kanto among the regions except Hokkaido (35.3 %).

Hokkaido has 84 % of the pastureland area in Japan [12, 13] and is located in the most northern part of the country with a relatively cool climate, making it the most suitable place to raise the Holstein breed. Therefore, there are more large cattle-breeding ranches in Hokkaido than in other parts of Japan [13]. On the contrary, dairy farms in other areas face difficulties managing both farm-size enlargement for cost efficiency and securing sufficient space for raising cattle. Thus, these round trips of dairy cows between Hokkaido and other parts of Japan seem to result from the outsourcing efforts of dairy farms outside Hokkaido.

In addition, among the pregnant heifers shipped from Hokkaido, 25 % of them returned from cattle-breeding ranches in Hokkaido to their original farms, as mentioned above, and 75 % were heifers born in Hokkaido. During FY2018, 67 % of Japanese dairy calves were born in Hokkaido (Supplemental Figure S3), which is higher than the proportion of dairy cows in Hokkaido relative to the total number of dairy cows in Japan (60 %), suggesting that Hokkaido supplies replacement dairy cows to other regions. In Japan, the production of the so-called F1 cattle, calves of a dairy dam with the semen of the Japanese Black breed, is popular on dairy farms because these calves are sold to beef feedlot farms at higher prices than purebred dairy calves. In 2019, the semen of the Japanese Black breed was used for 36 % of inseminated dairy cows [14]. This proportion is higher outside Hokkaido (50 %) than in Hokkaido (25 %), and as a result, the supply of replacement dairy cows outside Hokkaido cannot fulfill the self-demand in these areas and are instead covered by the supply from Hokkaido.

The observed intra-regional movements, whose proportion was higher than that of inter-regional movement similar to those in other countries [4, 5, 7], showed a seasonal variation; that is, more dairy cows moved in May and October than in other months (Fig. 4b and Additional file 1: Figure S5). The monthly age distribution at movement was different for May and October (Fig. 8). These results suggest two types of intra-regional movement: a constant movement throughout the year, including May and October, and the season-specific movement that occurred only in May and October. As 78 % of intra-regional movement occurs in Hokkaido (Fig. 2), these two trends seem to be mostly restricted to Hokkaido. In months other than May and October, three peaks were observed in the age at movement: less than 1 month, around 9 months, and around 22 months. This age trend at movement was generally similar throughout all months. Even in May and October, it seems that these three peaks were masked by additional season-specific movements. The first peak should represent the movement of newborn calves to nurturing facilities because the movement occurred shortly after birth, less than 1 month of age. As for the second and third peaks, since the ages of both peaks were similar to those observed in the above-mentioned inter-regional movement in Hokkaido, these movements could also be interpreted as the round-trip movement before and after rearing and breeding in cattle-breeding ranches from farms inside Hokkaido. In addition to the round-trip movement to and from cattle-breeding ranches, these two age groups also seemed to reflect the common ages of replacement dairy cows traded between dairy farms in Japan [15].

Regarding the seasonal movement observed only in May and October, the peaks had similar height, and age at movement differed by almost 6 months, being 14 months old in May and 20 months old in October (Fig. 8), similar to the difference between May and October. Our analysis of tracked individual cattle during FY2018 showed that 37 % of dairy cows moved in May were identical to 40 % of dairy cows moved in October. These results imply that many dairy cows, specifically growing heifers, moved in both May and October of the same year. In Hokkaido, summer grazing is customarily practiced. Generally, cows enter the common pasture around May, when pasture grass becomes available, and leave the land around October before the snowfall season. Since the average age of first calving for dairy cows in Japan is 24.7 months, the optimal age for breeding of Holsteins is approximately 14 months. As it is necessary to avoid calving during the grazing period, our results found that the heifers shipped for free-range grazing in May are pre-breeding heifers around 14 months of age, which will reach the appropriate age for breeding during the grazing period, and they leave the common pasture before calving. Seasonal cattle movement for grazing is also practiced in other countries located in alpine regions, such as Switzerland [16].

In this study, two characteristic round-trip movements of Japanese dairy cows were demonstrated: the non-seasonal movement of cattle breeding ranches in Hokkaido and the seasonal movement of heifers for summer grazing within Hokkaido. Cattle-breeding ranches and common pastures are typically facilities where large numbers of animals from multiple farms gather. They are, therefore, places where the risk of the introduction and spread of infectious diseases is high. Cattle breeding ranches are, in fact, reported as a risk factor for animal disease transmission in Japan and other countries [17, 18].

## Conclusions

This study revealed seasonal and regional heterogeneity in the movement patterns of dairy cows in Japan. The characteristics of the regions, seasons, and facilities where dairy cows are gathered will help develop effective disease control measures, such as testing animals at the time of entry and exit of facilities. In addition, information on cattle movement patterns is useful for developing models of infectious disease transmission between farms or regions and should be carefully analyzed and reflected in the model.

## Methods

### National database of cattle information and movement record

 In Japan, following the outbreak of BSE in September 2001, a traceability system for cattle was established, and a system based on “the law for special measures concerning the management and relay of information for individual identification of cattle” has been in place since December 2003. All cattle are obliged to carry an ear tag with a unique individual identification number within 7 days of birth, and all movements from birth to death (including slaughter) are registered and maintained in the national database called “Individual Cattle Identification Register (ICIR),” managed by the National Livestock Breeding Center (NLBC). In accordance with the law, everyone involved in cattle movement or slaughter, such as farmers, livestock markets, and slaughterhouses, should report the movement details (date, movement type such as birth, transfer, and slaughter, and facility number) to NLBC. In this study, cattle movement records, individual cattle information, and facility information, accumulated in ICIR for 14 years from FY2005 to FY2018, were obtained from NLBC and used for analysis. All data were directly delivered from NLBC to the National Institute of Animal Health under the condition of “the Regulation for the Second Use of Individual Cattle Identification Register of National Livestock Breeding Center.” All data were anonymized by replacing the farm and animal-identifiable data with randomized identification numbers before the analysis.

### Preparation of data for analysis

The cattle movement records were combined with individual and facility information using individual identification numbers and facility numbers. Inaccurate movement records of cattle (e.g., movement records after death or before birth) were deleted. For movement records with a stay of less than 1 day, the movements before and after the stay were combined (herewith, movements through markets were converted to between-farm movements). All records of dairy cows were extracted.

### Number of dairy cows and births

To understand the background to the movement of dairy cows, the number of dairy cows as of April 1, 2018, was summarized by region. Then, the records of “birth” were extracted, and the number of births during FY2018 was counted by region and month.

### Regional level movement

In this study, the records of “between-farm movements” were extracted by excluding “birth (including import)” and “death (including slaughter)” from the whole records of dairy cows. For the between-farm movement records, the departure and arrival farms were categorized into the following 7 regions: Hokkaido, Tohoku, Kanto, Chubu, Kinki, Chugoku/Shikoku, and Kyushu/Okinawa, as shown in Fig. 1; Table 1. The number of dairy cows that moved between farms was counted by the departure and arrival regions.

#### Age and month at the time of movement

For both inter- and intra-regional movements, the number of dairy cows was counted for each month of age at the time of movement and each month of movement. When the number of monthly movements showed the possibility of seasonality, time series analysis was conducted. As the time series analysis, STL decomposition (Seasonal-Trend decomposition based on Loess) [19] and seasonal subseries plotting was conducted.

All analysis was conducted using R version 3.5.3 [20] with the forecast package [21, 22] for time series analysis.

## Supplementary Information

**Additional file 1.**

## Abbreviations

BSE: Bovine spongiform encephalopathy
ICIR: Individual Cattle Identification Register
NLBC: National Livestock Breeding Center
