# Supplementary material for: Region-wise analysis of dairy cow movements in Japan
Source: BMC Vet Res. 2021 Sep 9;17:305. doi: 10.1186/s12917-021-03008-3 (PMC8428051; doi:10.1186/s12917-021-03008-3)
Supplement: Supplementary file 1 — Additional file 1. [file 12917_2021_3008_MOESM1_ESM.docx]

# Supplementary Material: Region-wise analysis of dairy cow movements in Japan

Yoshinori Murato^1^, Yoko Hayama^1^, Yumiko Shimizu^1^, Kotaro Sawai^1^, Emi Yamaguchi^1^, Takehisa Yamamoto^1*^

^1^Epidemiology Unit, National Institute of Animal Health, National Agriculture and Food Research Organization, Tsukuba, Ibaraki, Japan

^*^Corresponding author: Takehisa Yamamoto mtbook@affrc.go.jp

## SUPPLEMENTARY FIGURES


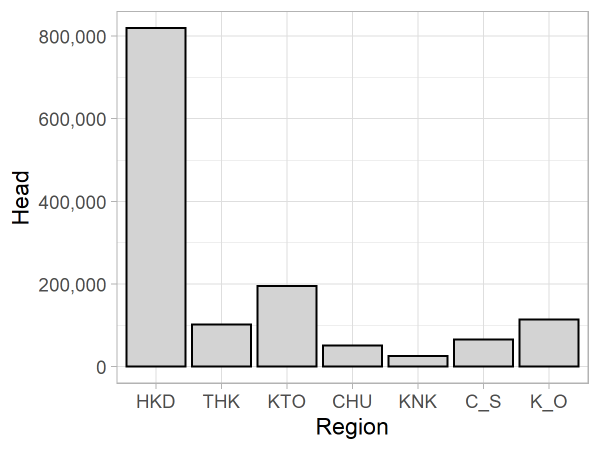


**Figure S1.** Number of dairy cows by region in Japan as of April 1, 2018.


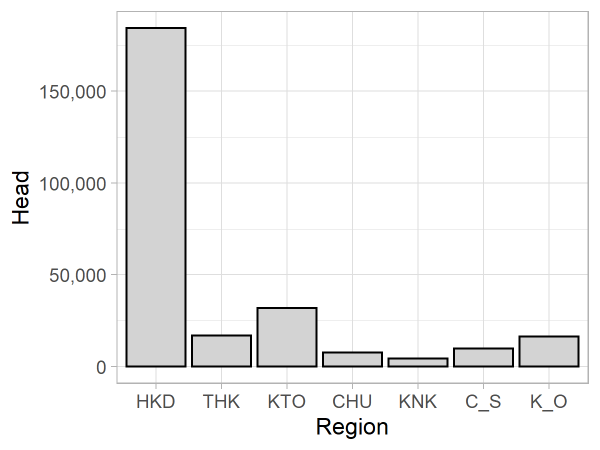


**Figure S2.** Number of dairy cow births by region in Japan in FY2018.


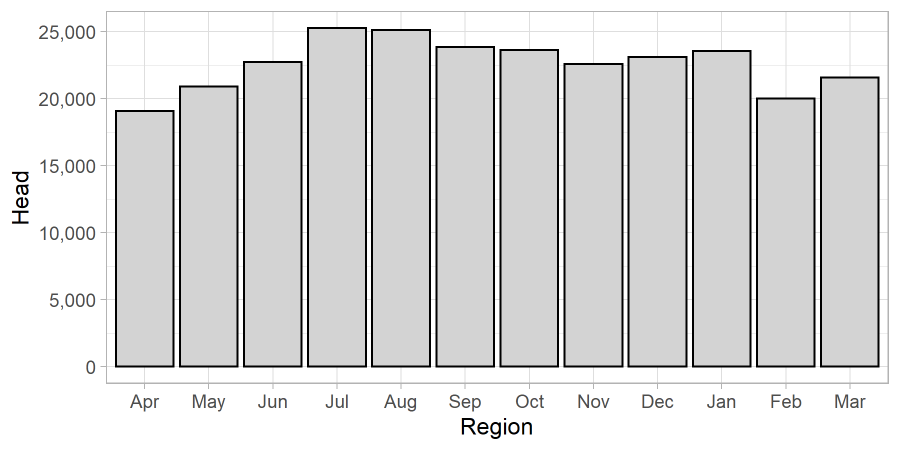


**Figure S3.** Number of dairy cow births by month in Japan in FY2018.


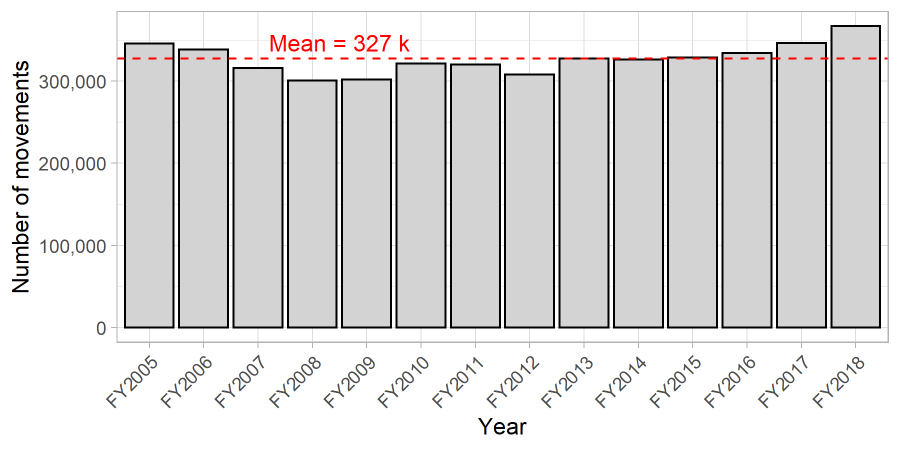
 **Figure S4.** Number of between-farm movements of dairy cows per fiscal year in Japan.

a**
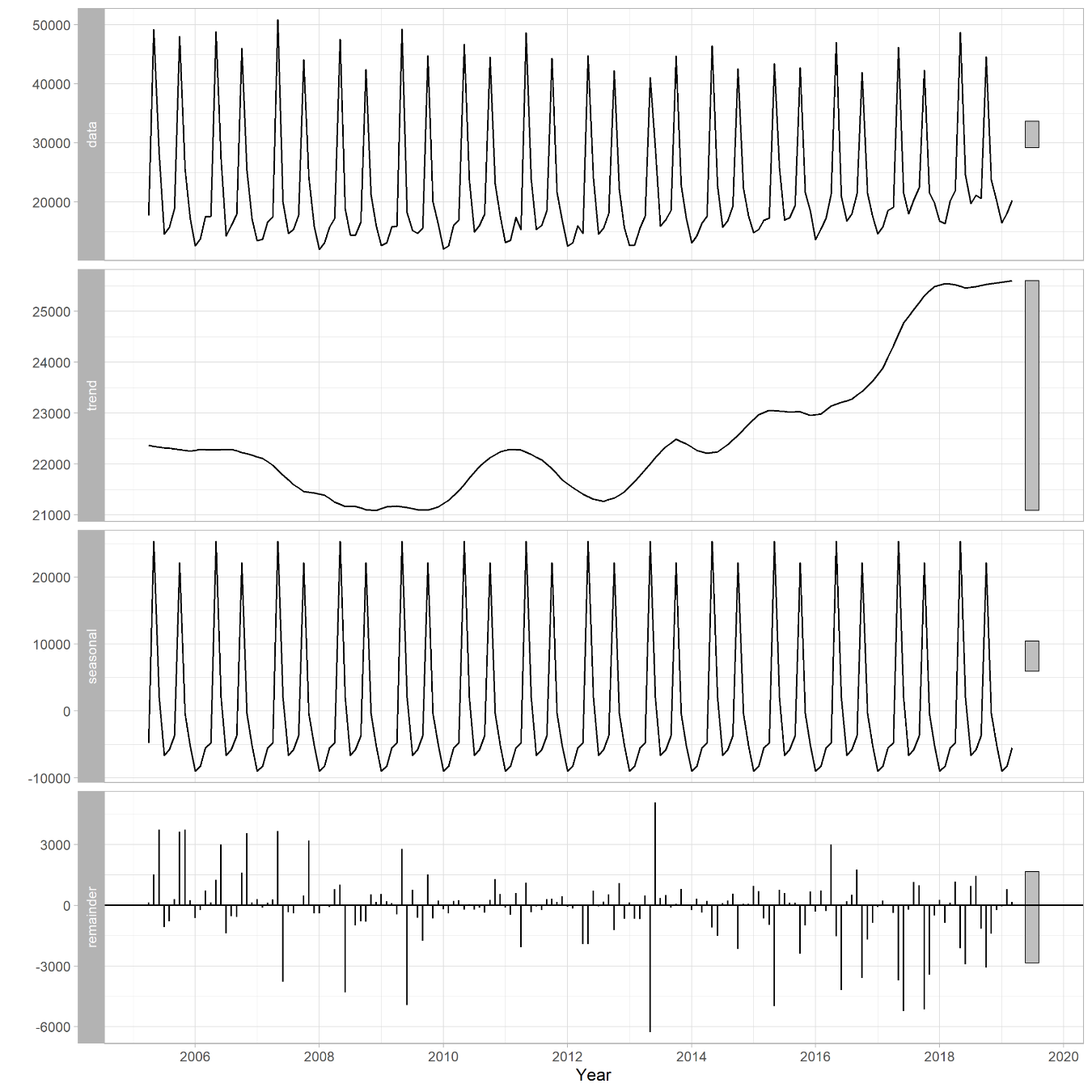
**

b


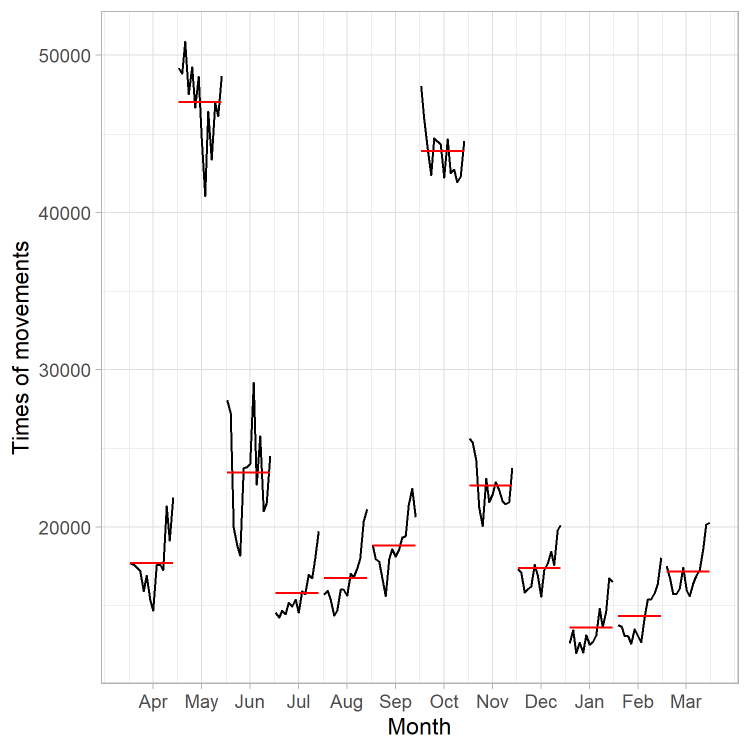


**Figure S5.** Time series analysis of monthly movements of dairy cows in intra-regional movement in Japan from FY2005 to FY2018.

Plot (a) indicates the original data (top), the estimated trend (second from top), the seasonal component (third from top), and the random component (bottom). Seasonal trend by months throughout the year is extracted as (b) by plotting monthly subseries plot.
